# Machine learning predictive performance evaluation of conventional and fuzzy radiomics in clinical cancer imaging cohorts

**DOI:** 10.1007/s00259-023-06127-1

**Published:** 2023-02-04

**Authors:** M. Grahovac, C. P. Spielvogel, D. Krajnc, B. Ecsedi, T. Traub-Weidinger, S. Rasul, K. Kluge, M. Zhao, X. Li, M. Hacker, A. Haug, Laszlo Papp

**Affiliations:** 1 Division of Nuclear Medicine, Medical University of Vienna, Vienna, Austria; 2 Christian Doppler Laboratory for Applied Metabolomics, Medical University of Vienna, Vienna, Austria; 3 Center for Medical Physics and Biomedical Engineering, Medical University of Vienna, Waehringer Guertel 18-20, AT-1090 Vienna, Austria; 4 Department of Nuclear Medicine, Peking University Third Hospital, Beijing, People’s Republic of China

**Keywords:** Machine learning, Radiomics, Fuzzy radiomics, PET, PET/CT, PET/MRI

## Abstract

**Background:**

Hybrid imaging became an instrumental part of medical imaging, particularly cancer imaging processes in clinical routine. To date, several radiomic and machine learning studies investigated the feasibility of in vivo tumor characterization with variable outcomes. This study aims to investigate the effect of recently proposed fuzzy radiomics and compare its predictive performance to conventional radiomics in cancer imaging cohorts. In addition, lesion vs. lesion+surrounding fuzzy and conventional radiomic analysis was conducted.

**Methods:**

Previously published 11C Methionine (MET) positron emission tomography (PET) glioma, 18F-FDG PET/computed tomography (CT) lung, and 68GA-PSMA-11 PET/magneto-resonance imaging (MRI) prostate cancer retrospective cohorts were included in the analysis to predict their respective clinical endpoints. Four delineation methods including manually defined reference binary (Ref-B), its smoothed, fuzzified version (Ref-F), as well as extended binary (Ext-B) and its fuzzified version (Ext-F) were incorporated to extract imaging biomarker standardization initiative (IBSI)-conform radiomic features from each cohort. Machine learning for the four delineation approaches was performed utilizing a Monte Carlo cross-validation scheme to estimate the predictive performance of the four delineation methods.

**Results:**

Reference fuzzy (Ref-F) delineation outperformed its binary delineation (Ref-B) counterpart in all cohorts within a volume range of 938–354987 mm^3^ with relative cross-validation area under the receiver operator characteristics curve (AUC) of  +4.7–10.4. Compared to Ref-B, the highest AUC performance difference was observed by the Ref-F delineation in the glioma cohort (Ref-F: 0.74 vs. Ref-B: 0.70) and in the prostate cohort by Ref-F and Ext-F (Ref-F: 0.84, Ext-F: 0.86 vs. Ref-B: 0.80). In addition, fuzzy radiomics decreased feature redundancy by approx. 20%.

**Conclusions:**

Fuzzy radiomics has the potential to increase predictive performance particularly in small lesion sizes compared to conventional binary radiomics in PET. We hypothesize that this effect is due to the ability of fuzzy radiomics to model partial volume effects and delineation uncertainties at small lesion boundaries. In addition, we consider that the lower redundancy of fuzzy radiomic features supports the identification of imaging biomarkers in future studies. Future studies shall consider systematically analyzing lesions and their surroundings with fuzzy and binary radiomics.

**Supplementary Information:**

The online version contains supplementary material available at 10.1007/s00259-023-06127-1.

## Introduction

Cancer is one of the leading causes of death worldwide [1]. Medical imaging became an instrumental part of cancer detection, with positron emission tomography (PET) playing an important role in the imaging of metabolic activities, and the characterization of tumor heterogeneity in vivo [2]. Hybrid imaging systems relying on PET/computed tomography (CT) are considered the gold standard of cancer imaging, while PET/Magnetic resonance imaging (MRI) is in the process of wide-scale adoption worldwide [3, 4].

While imaging is routinely employed to detect tumors, to date, it is mainly used for visual inspection and for basic imaging biomarker measurements such as metabolic tumor volume [5]. In contrast, various studies proposed the utilization of radiomics—the approach to extract different imaging features from tumors—for analysis [6, 7]. Contrary to promising results, radiomic studies had been challenged by their poor reproducibility due to various factors such as biological and imaging differences, delineation, radiomic feature extraction equation variations as well as their parameters (e.g., resolution, bin size, binning method) [7, 8]. An important consolidation phase had been started with the proposal of the Imaging Biomarker Standardization Initiative (IBSI) [9]. However, IBSI does not cover all important aspects of radiomics such as delineation, which has a profound effect on radiomic features [2, 10–12] and it appears to be mainly affected by multi-observer variabilities and cohort, as well as imaging characteristics [10]. Consequently, delineation of tumor lesions had been extensively investigated in the corresponding literature [10, 13]. While various tumor delineation approaches had been proposed [14–16], certain studies looked into analyzing not only the lesions, but also their surroundings [17, 18]. Recently, deep learning (DL) has been demonstrated as a powerful technique to delineate suspicious lesions in PET for subsequent analysis with e.g., radiomics and machine learning (ML) [13, 19–22]. However, the common property of all the above approaches is that they result in a binary delineation mask or volume of interest (VOI) for radiomic analysis, meaning, that a particular PET voxel is either part of the analysis or not, regardless of how certain its membership in the given VOI is. This approach has various drawbacks. First, operating with binary masks renders the radiomic analysis sensitive to PET partial volume effects (PVE) especially at lesion boundaries [23]. Second, delineation errors may result in suboptimal radiomic analysis at lesion boundaries, regardless of PVE, and third, multi-observer variations result in different radiomic outcomes, which makes the repeatability of reported studies challenging [10, 13, 16]. Fuzzy radiomics had been presented as a potential approach to handle voxel membership uncertainties by relying on non-binary probability masks [24]. However, to date, it has not been utilized and evaluated in real clinical cancer settings.

In theory, fuzzy radiomics has numerous advantages. First, it can model and encompass PVE in the given mask as to the properties of the given imaging system. Second, it can also encode multiple observer’s delineations as a weighted mask. Third, it can consider not just the given lesion, but its surroundings with appropriate weights for the analysis. Last, it can directly handle DL delineation masks that are inherently probabilistic, but are routinely post-processed and dichotomized by a threshold to provide a binary mask for subsequent analysis [19, 20, 22, 25].

In light of the above, the aim of this study was to compare the effect of binary and fuzzy delineation masks in both lesions and their surroundings, through investigating the performance of ML prediction models built in various cancer cohorts to predict their clinical endpoints. Specifically, this study had the following objectives: (a) to collect various cancer imaging cohorts having different characteristics regarding the imaging systems and PET tracers involved; (b) to perform classic and fuzzy radiomic feature extraction relying on binary and fuzzy probability masks of lesions as well as their surroundings; and (c) to compare ML performance of predicting cohort-specific clinical endpoints relying on the above feature extraction approaches.

## Methods

### Cohorts

This study relied on already delineated lesions from three retrospectively available cancer imaging cohorts including 11C methionine (MET) PET glioma, 18F-FDG PET/CT lung and 68GA-PSMA-11 PET/MRI prostate cancer cases. For details of how these delineations were done, see Sec. Delineation. All cohorts had been previously investigated and presented in various ML studies [26–30] with follow-up up to 3 years. This study included lesions from the above cohort databases that fulfilled the minimum 64 voxel number constraint [31], resulting in 105, 543 and 121 delineated lesions in glioma, lung and prostate cases respectively.

**Fig. 1:**
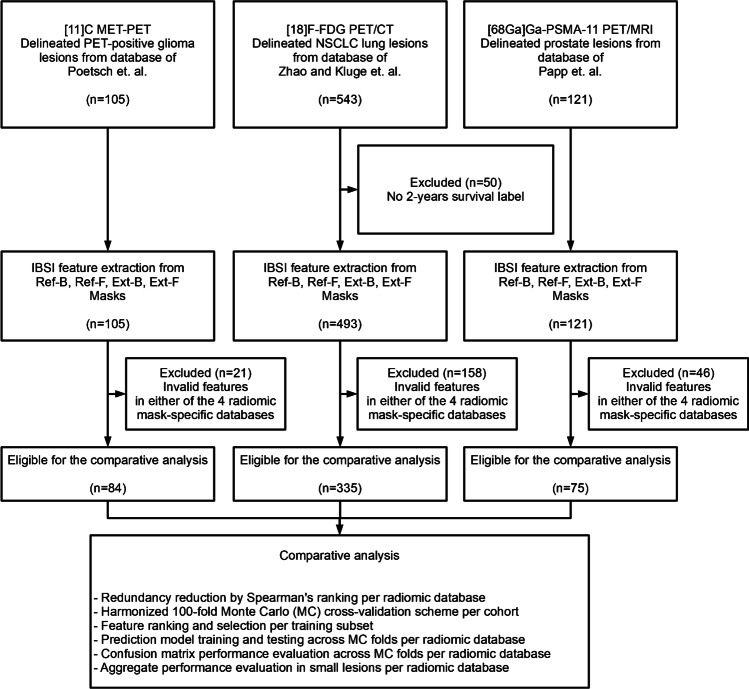
The CONSORT diagram of preparing and analyzing the cohorts of this study. Already delineated glioma, lung and prostate lesions (Ref-B) were collected from databases that were analyzed and published in prior studies [27–29]. Glioma and lung cohorts had one lesion per patient, while the prostate cohort contained multiple lesions per patient. Reference labels for glioma, lung and prostate lesions were 3-years survival, 2-years survival and low-vs-high risk, respectively. Three additional delineations were generated form Ref-B delineations: a fuzzy mask (Ref-F), an extended binary mask (Ext-B) and an extended fuzzy (Ext-F) mask. Fuzzy masks were generated by a three-dimensional Gaussian filtering, relying on the physical resolution of each cohort's PET imaging system. Samples having no variations or invalid radiomic values (e.g., by low-uptake lesions) were excluded from all delineation-specific databases to ensure a harmonized comparison among them. The resulted four databases per cohort underwent the comparative analysis (see the “Methods” section)

All cohorts had been approved for analysis by their respective institutional review boards and the need for informed consent was waived in retrospective studies. The clinical endpoints were 3-years survival, 2-years survival and low-vs-high risk in glioma, lung and prostate cohorts, respectively. See Supplemental: Patient Cohorts and Supplemental Table S1-S3 for patient and clinical characteristics of the utilized cohorts. See Fig. 1 for the CONSORT diagram of this study.

### Delineation

This study did not intend to promote a new delineation approach, but intended to compare binary vs. fuzzy masks of lesions as well as their surroundings regarding their performance in predicting clinical endpoints. Therefore, this study included four delineation approaches to compare in each cohort, where the existing delineation of each cohort was serving as reference binary (Ref-B) mask provided by clinical experts in consensus. The number of clinicians involved in this step was cohort-specific (See Supplemental: Table S4).

**Fig. 2:**
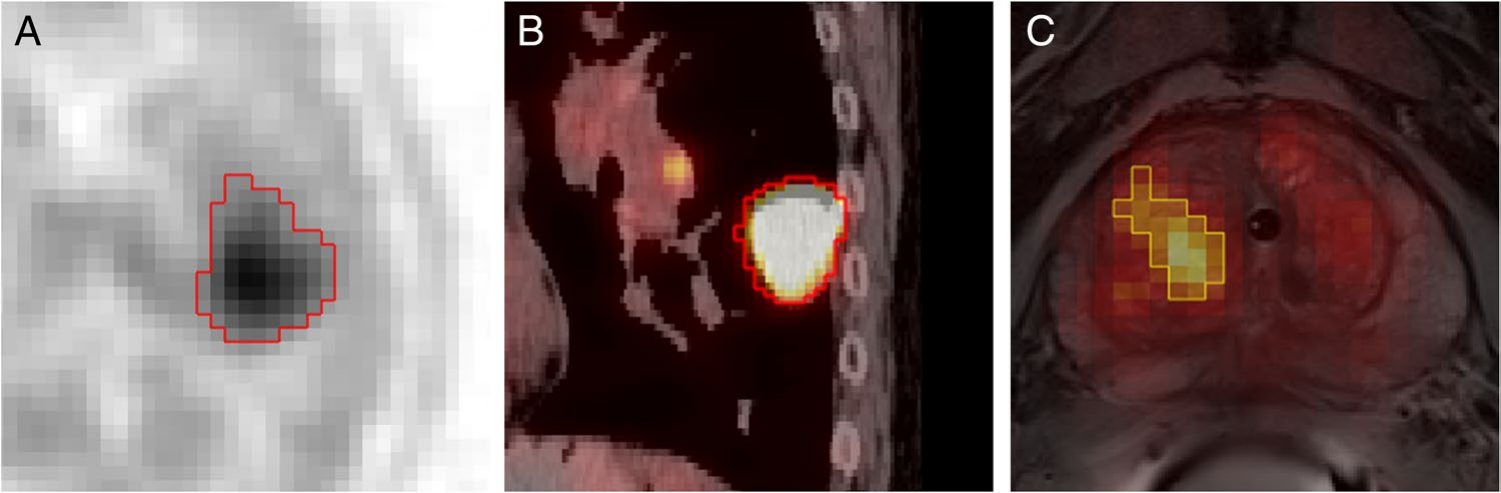
Manual delineation (Ref-B) example views of the cohorts involved in this study. **A** Glioma tumor (axial view, inverted gray palette with range 0–7 SUV); **B** Lung tumor (sagittal view, PET hot metal palette with range 0–5 SUV, CT gray palette with range -100.200 HU); **C** Prostate tumor (oblique-axial view, PET hot iron palette with range 0–9 SUV, T2w MRI grey palette). PET—Positron Emission Tomography; SUV—Standard Uptake Value; T2w—T2-weighted magnetic resonance imaging (MRI)

The delineation of cohort-specific lesions was originally performed in the Hermes Hybrid 3D software ver. 4.0.0 (Hermes Medical Solutions, Stockholm, Sweden) relying on standard three-dimensional (3D) iso-count VOIs [26, 27, 32]. Where needed, manual slice-by-slice modifications were performed to result in the final Ref-B VOI. See Fig. 2 for example screenshots of the delineation in each cohort. In addition to the above, a reference background region for tumor-to-background ratio (TBR) normalization of PET images was also available for each case (see Supplemental Table S4 for details).

**Fig. 3:**
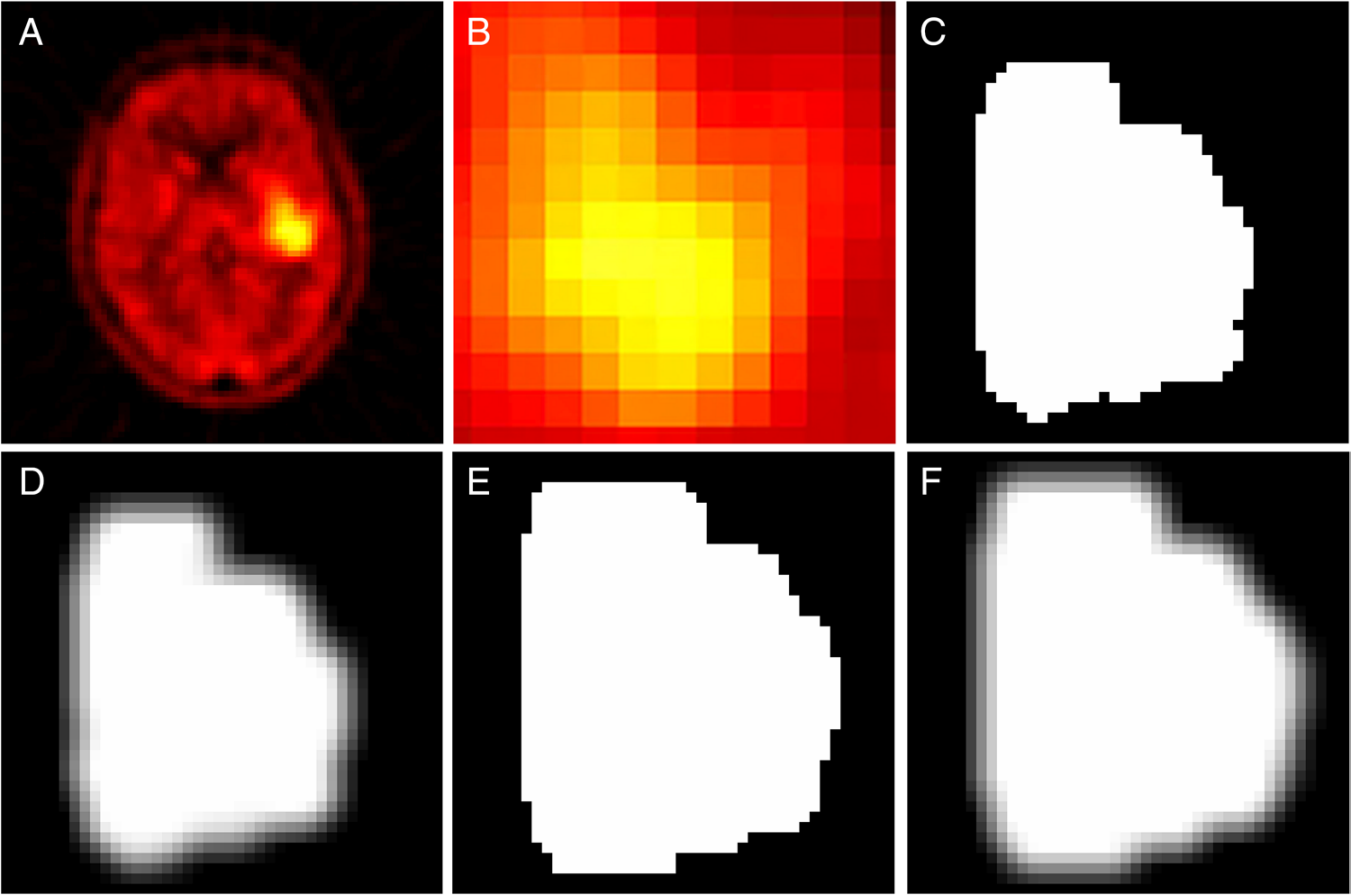
**A** Axial view of an example lesion in the Glioma cohort (MET-PET, Hot iron palette with palette ranges 0–6 SUV). **B** Magnified axial view of the tumor **C** Reference binary delineation (Ref-B). **D** Reference fuzzy delineation (Ref-F). **E** Extended binary delineation (Ext-B). **F** Extended Fuzzy delineation (Ext-F). MET—methionine; PET—positron emission tomography; SUV—standard uptake value. Note that images C-F are delineated masks with 2 × 2 × 2 mm resampling for the radiomic analysis

Based on the Ref-B VOI of each case, three additional delineation masks were generated: a reference fuzzy mask, by smoothing Ref-B by a 3D Gaussian filter [28] with a cohort-specific full-width half-maximum (FWHM), which corresponded to the physical resolution of the given imaging system in 3D (Ref-F); an extended binary mask by morphological dilating [33] Ref-B with 3 voxels in 3D (Ext-B), and an extended fuzzy mask by smoothing Ext-B mask with a 3D Gaussian kernel by applying the cohort-specific FWHM as smoothing parameter (Ext-F). The Gaussian FWHM for the 3D smoothing in case of both the reference (Ref-F) and extended (Ext-F) fuzzy mask generations were 5 mm, 4.7 mm, and 4.6 mm for glioma, lung and prostate cohorts respectively, according to the manufacturer-reported physical resolutions of their imaging systems (see Supplemental Table S4). For a visual comparison of an example lesion as well as the four masks, see Fig. 3.

### Feature extraction and normalization

All extracted VOIs were resampled to 2 × 2 × 2 mm voxel resolution by using Kriging interpolation in 3D [26, 34] and the PET standardized uptake values (SUV) were normalized by the mean of their respective background regions (See Supplemental Table S4 for details). Each TBR PET lesion VOI was subject to radiomic feature extraction following the IBSI guidelines where only features of “very strong” and “strong” multi-centric consensus as of the IBSI [9] were extracted. The above steps were performed for each of the four delineation masks (Ref-B, Ref-F, Ext-B and Ext-F) and resulted in 153 features per sample. In case either of the four delineation-specific radiomics database had invalid numbers or numbers with no variations (e.g., due to too small uptake in lesions in relation to the bin width), the given sample was removed from all four databases. This was necessary to ensure that the harmonized cross-validation split configuration had identical train-test split samples for all four delineation processes for comparison. This step resulted in 84, 335, and 75 samples in glioma, lung and the prostate cohorts, respectively (see Fig. 1).

Due to the properties of fuzzy radiomics, its feature calculation equations are identical to those defined by IBSI. The only difference between fuzzy and binary radiomics is that fuzzy radiomics takes weights with any value between 0.0 and 1.0 into account when calculating intermediate metadata e.g., textural matrices [6]. In contrast, classic radiomics consider that the weight of a voxel is 1.0 if it is part of the given delineation mask, and 0.0 otherwise. Therefore, classic radiomics is a special variant of fuzzy radiomics in case the mask only has 0.0 and 1.0 values (See Supplemental: Fuzzy vs. Classic Radiomics for details). As of this relationship, this study only utilized the IBSI-validated fuzzy radiomics engine to calculate both binary (Ref-B and Ext-B) and fuzzy (Ref-F and Ext-F) radiomic values for further analysis (See Supplemental Table S4 for details).

### Feature redundancy reduction

Since radiomic features are generally highly-redundant [35, 36], this study performed redundancy reduction (RR) in each of the four radiomic data tables corresponding to the four delineation methods of each cohort. The RR was performed by correlation matrix analysis with Spearman correlation coefficient 0.9 as threshold to identify redundant feature clusters [37]. From each redundant cluster, all features except the one with the highest variance were deleted per delineation type in each cohort.

### Harmonized cross-validation scheme and data preprocessing

Hundred fold Monte Carlo (MC) cross validation with 90–10% train-test ratio was utilized to generate training-test subsets per cohort [32]. While the splitting was random, only unique folds were allowed to be generated, and no lesions from the same patient were allowed to be part of a train-test split at once, to avoid patient-level data leakage. This step was necessary for the prostate cohort, where patients had multiple lesions [27]. The fold split configuration was harmonized within the given cohort for all four delineation-specific radiomic datasets to avoid split-specific predictive performance variations during the performance evaluation step of the study. The 10% test ratio was calculated for the minority subgroup of each cohort, followed by selecting equal number of test samples according to this ratio in order to ensure that each prediction label subgroup had the same test subgroup count [32]. This way, each training subset remained imbalanced. To handle class imbalance, Synthetic Minority Oversampling Technique (SMOTE) was utilized to obtain equal numbers of subgroups in each training subset [38]. In order to remain in the original IBSI feature space for supporting imaging biomarker identification and analysis, this study did not employ dimensionality reduction [39], but feature selection. Feature selection in the training subset was performed by *R*-squared ranking [32] where the number of selected features *f* was calculated by Eq. 1:

$$f=\sqrt{0.9*2*M}$$

where *M* represents the number of samples in the majority group within the given dataset. Since each cohort had a binary label to predict and the training subset ratio was 90%, 0.9 * 2 multiplier ensured that the number of features selected followed the curse of dimensionality rule [40] in relation to the number of samples in the preprocessed, SMOTE-extended training subset of each MC fold. See Table 1 for details of the collected cohorts in relation to sample counts and class imbalance ratios.

**Table 1:** Imaging modalities, sample counts and clinical endpoints to predict in the collected cohorts of this study. For clinical and patient characteristics of each cohort, see Supplemental Table S1-S3. Original sample counts refer to the number of cases this study incorporated to its analysis. Harmonized sample counts refer to the number of samples that were mutually-present as valid across all four delineation-specific radiomic databases. Class imbalance ratio refers to the ratio of the minority subclass vs. the number of all samples in the harmonized radiomic datasets

Cohort	Imaging	Sample count (original)	Sample count (harmonized)	Class imbalance ratio (harmonized)	To predict
Glioma	^11^C-MET PET	105	84	39%	3-years survival
Lung	^18^F-FDG PET/CT	543	335	39%	2-years survival
Prostate	^68^GA-PSMA PET/MRI	121	75	40%	Low-vs-high risk

### Prediction models

To minimize method-specific bias and the effect of bias-variance trade-off [41], mixed ensemble learning consisting of four different Random Forest (RF) [32, 42] and one multi-Gaussian (MG) [26] classifiers was built by analyzing each training subset of the given MC fold and for all four delineation-specific datasets (see Supplemental: Table S5 for parameters of the ML approaches). Each model predicted its cohort-specific binary label as of Table 1. The final prediction for a given input sample was provided as the majority vote of all RF and MG model instances. The above approach also allowed to eliminate the effects of hyperparameter variations when comparing the four delineation approaches across a harmonized cross-validation scheme, as the five model instances operated with a fixed hyperparameter set.

### Performance evaluation

The number of true positive (TP), true negative (TN), false positive (FP), and false negative (FN) cases were calculated across the test subsets of each ML fold utilizing their respective ML models. Confusion matrix analytics including sensitivity (SNS), specificity (SPC), positive predictive value (PPV), negative predictive value (NPV), accuracy (ACC), and area under the receiver operator characteristics curve (AUC) were calculated for each of the four delineation-specific radiomic models in each cohort. The 100-fold cross-validation AUC values in-between Ref-B vs. Ref-F, Ext-B and Ext-F were correlated by ANOVA analysis where *p* < 0.05 was considered as significance threshold to reject the null hypothesis. In addition, cross-validation AUC confidence intervals (CI) with 95% confidence ranges were calculated for each ML prediction model.

Further to the above, aggregate performance increase analysis of the four delineation methods across the cohorts within a standardized volume range of 938–354987 mm^3^ (or approx. 117–4500 voxels per lesion respectively, with 2.0 mm uniform voxel resolution for IBSI analysis—See Supplemental Table S4 for details) was performed. This step categorized volumes into 10 percentile clusters and the number of correct classifications for each delineation-specific predictive model were calculated per percentile cluster.

## Results

### Effect of delineation on feature redundancy

A consistent pattern across the different delineation methods (Ref-B, Ref-F, Ext-B and Ext-F) was identified regarding their effect on feature redundancy. As such, fuzzy radiomics decreased feature redundancy after performing RR compared to binary radiomics (see Table 2). The highest non-redundant feature count was identified in the prostate group for Ext-F (*n* = 52) compared to Ref-B (*n* = 35) delineations. For the list of high-ranking features per delineation approach in each cohort, see Supplemental: Feature Ranking.

**Table 2:** Number of IBSI radiomic features per cohort and per delineation method across feature extraction and data preprocessing steps including redundancy reduction and feature selection. Note that the number of IBSI features per-image extracted was 153 (See Supplemental Table S4); however, features having no variation or having invalid values (e.g., due to low uptake in the given lesion) were removed from the original IBSI features across all four delineation-specific datasets to ensure a unified comparison. *IBSI* imaging biomarker standardization initiative; *Ref-B* reference binary; *Ref-F* reference fuzzy; *Ext-B* extended binary; *Ext-F* extended fuzzy delineation

Cohort	Delineation method	#Features (original)	#Features (Redundancy reduced)	#Features (Ranked and selected)
Glioma	Ref-B	153	31	10
	Ref-F		39	10
	Ext-B		35	10
	Ext-F		41	10
Lung	Ref-B	153	24	20
	Ref-F		36	20
	Ext-B		23	20
	Ext-F		34	20
Prostate	Ref-B	153	35	9
	Ref-F		42	9
	Ext-B		35	9
	Ext-F		52	9

### Effect of delineation on feature ranking and selection

**Fig. 4:**
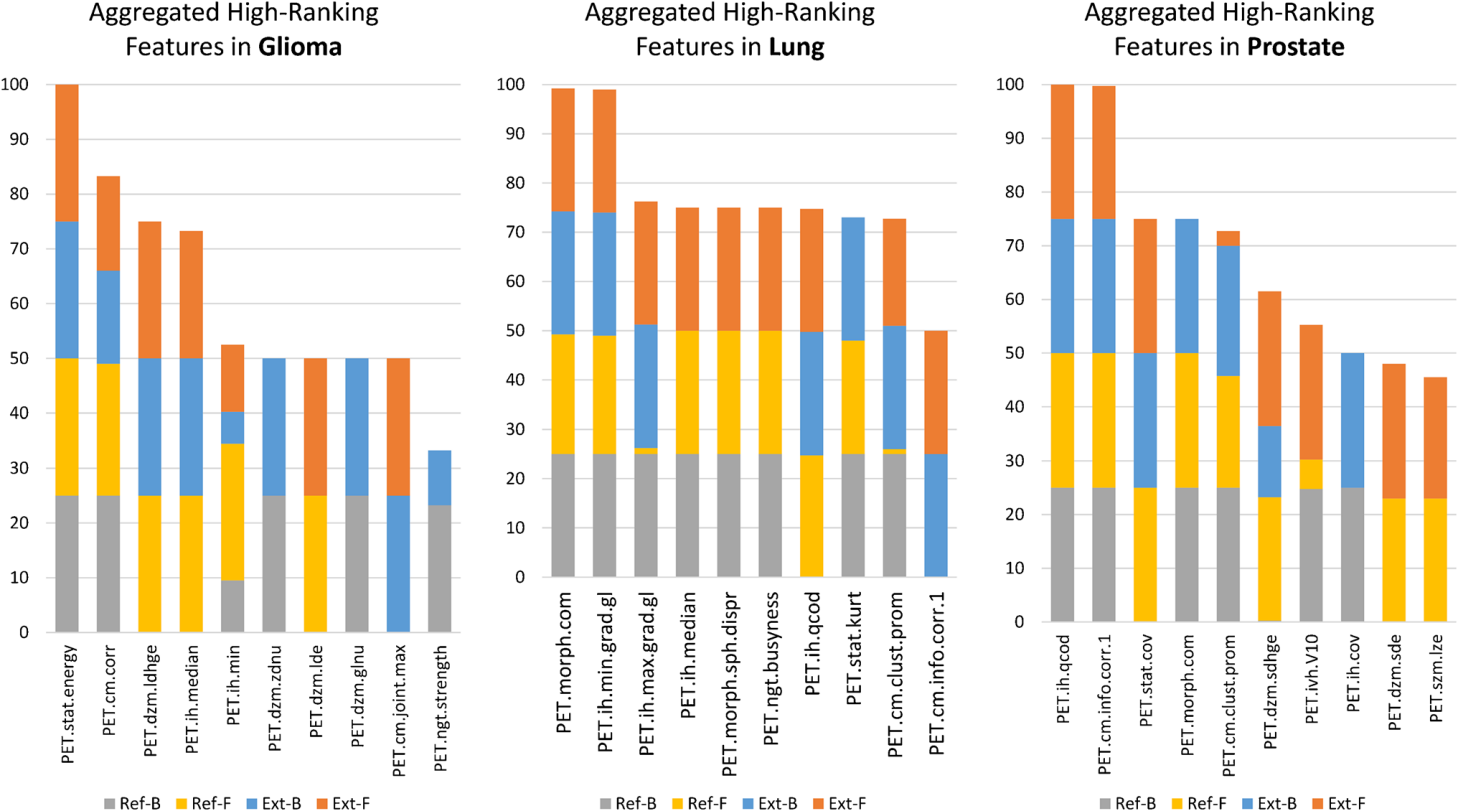
Aggregated high-ranking features in glioma (**A**), lung (**B**), and prostate (**C**) cohorts across radiomics features extracted by four delineation approaches utilized. Feature names are composed of the modality (PET, CT) type, followed by the IBSI-standard feature identified [9]. Feature occurrences (y-axis) are in the range of 0–100 with units of %. Ext-B—extended binary; Ref-B—reference binary; Ext-F—extended fuzzy; Ref-F—reference fuzzy delineation

High-ranking feature distributions accumulated across the four delineation types per cohort are shown in Fig. 4. In all cohorts, the highest-ranking aggregate features were also selected as high-ranking across all four delineation types in all cohorts (Fig. 4). Across all cohorts, two features were selected as high-ranking across all four delineations. Three, seven and three additional features were present in three delineation types as high-ranking in glioma, lung and prostate cohorts, respectively.

The per-delineation feature rankings (Supplemental: Feature Ranking), demonstrated a diverse distribution of feature importance across cohorts. Nevertheless, delineations that resulted in high predictive performance also tended to have a more-balanced feature rank distribution compared to those that had a skewed feature ranking distribution (Table 3).

**Table 3:**
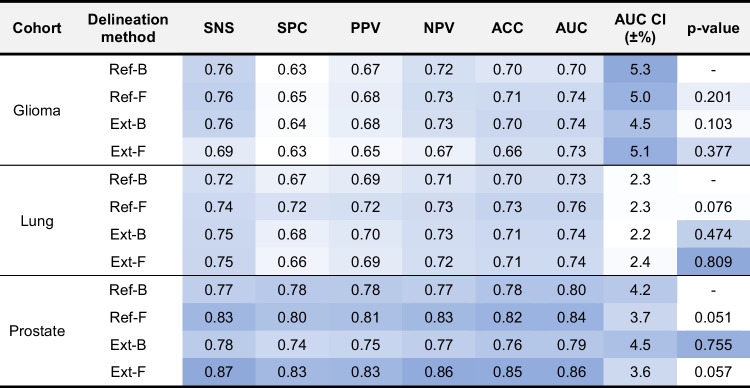
Performance values for glioma, lung and prostate cohorts relying on four delineation approaches to evaluate test cases within the harmonized 100-fold Monte Carlo cross-validation scheme. *P*-values represent the ANOVA analysis results in-between the reference binary delineation (Ref-B) and the other three delineation methods across the cross-validation area under the receiver operator characteristics curves (AUC). *SNS* Sensitivity; *SPC* specificity; *PPV* positive predictive value; *NPV* negative predictive value; *ACC* accuracy; *CI* confidence interval; *Ext-B* extended binary; *Ref-B* reference binary; *Ext-F* extended fuzzy; *Ref-F* reference fuzzy delineation. Color scale is normalized between the lowest (white) and the highest (blue) values in each category, where SNS – AUC, AUC CI (±%) and *p*-value form their own categories. Note that since the test subsets in the cross-validation scheme were balanced and the training subsets underwent class imbalance correction, ACC values reflect on a balanced classifier and they are in line with balanced accuracy (a.k.a. the average SNS and SPC)

### Performance evaluation

**Fig. 5:**
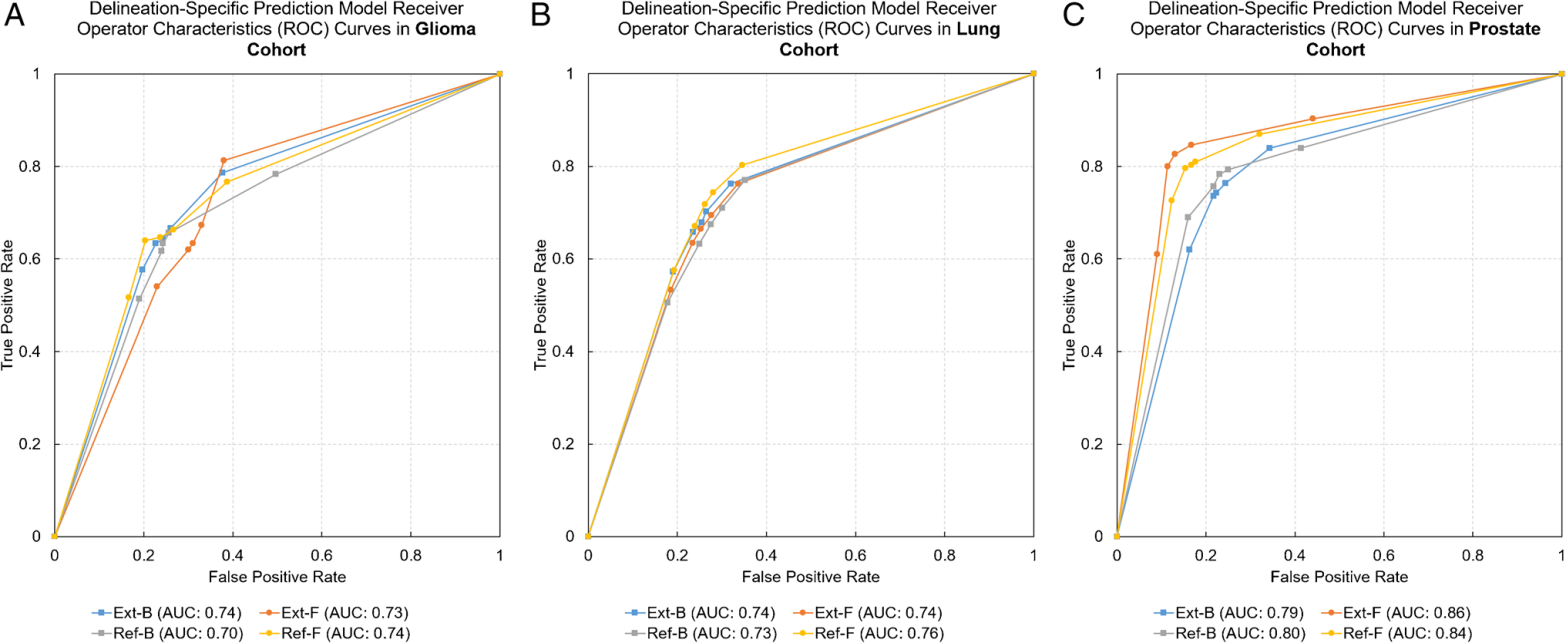
Receiver operator characteristics (ROC) curves of cohort-specific prediction models as of their respective delineation methods. **A** Glioma cohort. **B** Lung cohort. **C** Prostate cohort. Ext-B—extended binary; Ref-B—reference binary; Ext-F—extended fuzzy; Ref-F—reference fuzzy delineation; AUC—area under the ROC curve

Predicting 3-year survival in the glioma cohort was highest with the Ref-F delineation (AUC: 0.74, ACC: 0.71) vs. all other approaches (AUC: 0.70–0.74, ACC: 0–66-0.70). Similarly, predicting 2-year survival in the lung cohort demonstrated the highest performance with the Ref-F delineation (AUC: 0.76, ACC: 0.73) compared to other approaches (AUC: 0.73–0.74, ACC: 0–70-0.71). Predicting low-vs-high risk in the prostate cohort yielded the highest performance by utilizing Ext-F (AUC: 0.86, ACC: 0.85) and Ref-F (AUC: 0.84, ACC: 0.82) compared to the other approaches (AUC: 0.79–0.80, ACC: 0.78–0.76). See Fig. 5 for the receiver operator characteristics (ROC) curve comparisons of each of the four delineation approaches in the cohorts. See Table 3 for the detailed test confusion matrix results of the four delineation methods in each cohort. ANOVA p-value analyses revealed that the null hypothesis of performance values having no differences cannot be rejected (*p*-value ranges 0.051–0.809). Nevertheless, in case of prostate, *p* = 0.051 between Ref-B vs. Ref-F and *p* = 0.057 between Ref-B vs. Ext-F were near the significance level. In these cases, the performance values were demonstrating the highest differences as well (see Table 3). A similar pattern was visible in case of lung with *p* = 0.076 between Ref-B vs. Ref-F.

**Fig. 6:**
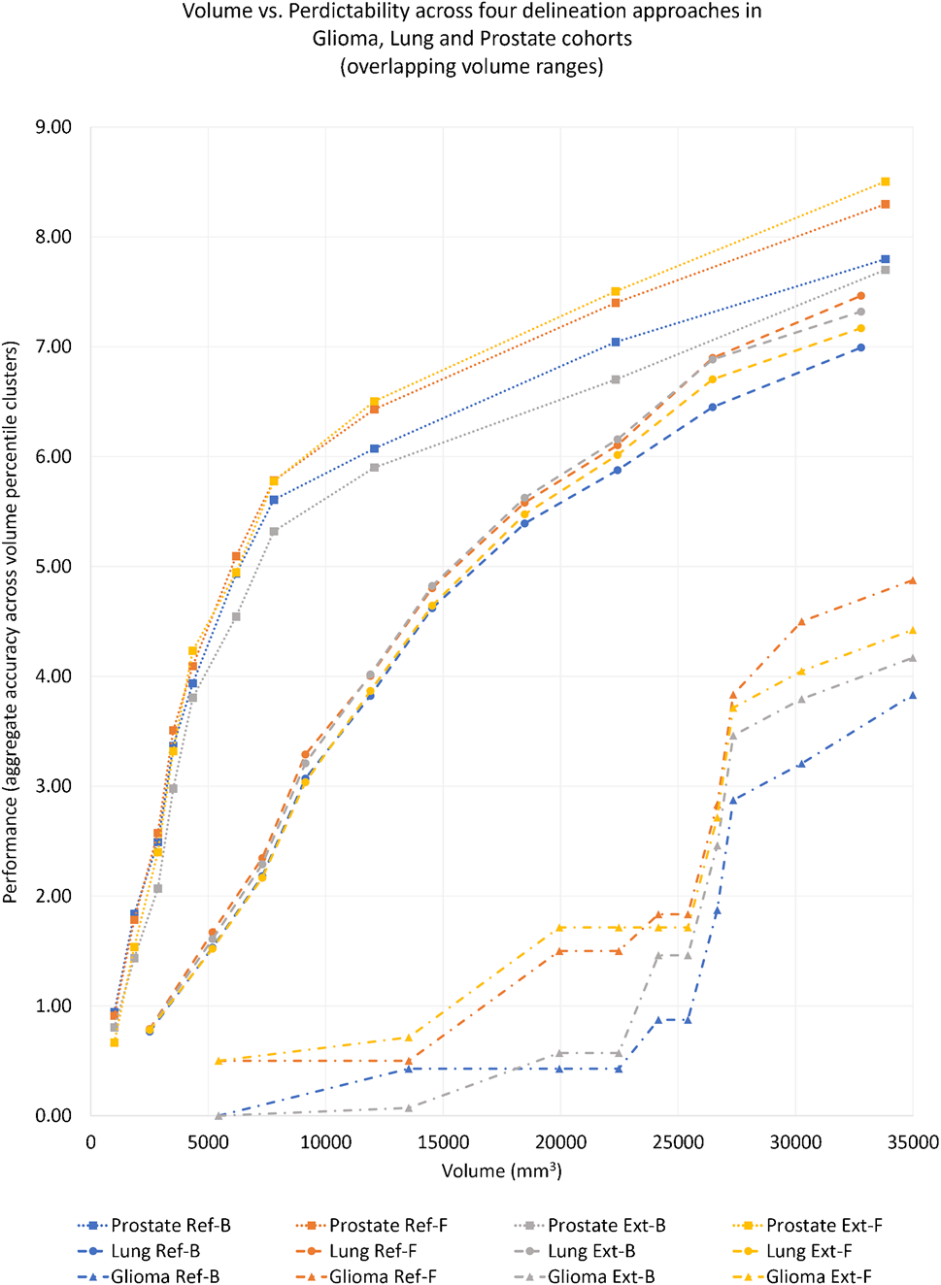
Aggregate performance increase of the four delineation types (Ref-B, Ref-F, Ext-B, Ext-F) across glioma, lung and prostate cohorts categorized to 10 percentile volume clusters within the common volume range of 938–354,987 mm^3^ or approx. 117–4500 voxels per lesion respectively. Ext-B—extended binary; Ref-B—reference binary; Ext-F—extended fuzzy; Ref-F—reference fuzzy delineation

Aggregated performance increase across volume percentiles revealed that reference fuzzy (Ref-F) delineations outperformed reference binary (Ref-B) delineations within a common volume range across glioma, lung and prostate cohorts with an aggregate AUC of  +4.7–10.4. (see Fig. 6). Nevertheless, the least significant differences among the four delineation approaches were present in the lung cohort, which was in line with the overall performance variations in this cohort when considering all its volume ranges (Table 3). The glioma cohort demonstrated that the advantage of fuzzy (Ref-F, Ext-F) delineations over binary ones (Ref-B, Ext-B) was already present in small lesions (~5000 mm^3^,  ~625 voxel count) and further increased afterwards. The prostate cohort revealed that the advantage of fuzzy delineations – Ext-F in particular – became prominent at approx. 8000 mm^3^ or  ~1000 voxel counts within lesions.

## Discussion

The effect of delineation on radiomic analyses in PET has been extensively investigated. To date, reports regarding reproducibility of predictive performance across various delineation approaches have been inconclusive and often cohort-specific [10, 43]. This study investigated the effect of conventional binary as well as fuzzy radiomics in both lesions and their surroundings by predicting clinically relevant endpoints in PET and hybrid imaging cancer cohorts. Across all cohorts, reference fuzzy (Ref-F) delineations outperformed reference binary (Ref-B) delineations with 3–4% AUC. We consider that this phenomenon is due to the fact that our fuzzy masks were specific to the given PET imaging system's physical resolutions, which allowed the modeling of partial volume effects (PVE) directly in the radiomics calculations. While the ANOVA p-value analysis could not reject the null hypothesis (a.k.a. performance values have no differences), cohort-specific predictive performance variations demonstrated a diverse pattern.

Specifically, the glioma MET-PET cohort had the highest AUC (0.74) and ACC (0.71) with the reference fuzzy (Ref-F) radiomics to predict 3-year survival. The second highest AUC (0.74) and ACC (0.70) was achieved by the and extended binary (Ext-B) delineation. We hypothesize that the relevance of Ext-B in this cohort is due to the infiltrating behavior of glioma [29, 44], which can be better characterized by employing extended binary radiomics.

In the lung 18F-FDG PET/CT cohort, Ext-B and fuzzy (Ext-F) delineations slightly increased predictive performance of 2-year survival, however, the highest performance increase was identifiable with the Ref-F delineation (Ref-F AUC: 0.76 vs. Ref-B AUC: 0.73). Since the PET acquisitions utilized no motion compensation, the reconstructed PET were subjects to motion artefacts [45–48]. Therefore, we argue that the reference delineations were already subjects to overestimation of the true metabolic tumor volumes in this cohort. Consistently, further extending the reference could not significantly increase predictive performance (*p* = 0.474–0.809). This implies that fuzzy radiomics may not be able to counter-balance motion artefact-related smoothing effects, which is logical, as motion may significantly alter the heterogeneity pattern within tumors, not only at the tumor boundaries [48].

Contrary to the above, the 68GA PSMA-11 PET/MRI study yielded the highest AUC of 0.86 with Ext-F, followed by the AUC of 0.84 with Ref-F delineation against binary delineations (Ext-B AUC: 0.79, Ref-B AUC: 0.80). While the generic superiority of reference fuzzy delineations was consistently demonstrated in this study, the highest performance of Ext-F delineation is considered to be due to the lowest feature redundancy achieved with this delineation. Specifically, the Ref-B delineation resulted in 35 non-redundant features before feature ranking and selection. In contrast, Ref-F and Ext-F resulted in 42 and 52 non-redundant features, respectively. Having a higher number of non-redundant features supports the identification of more high-ranking features, thus, may potentially yield high-performing models. Overall, the highest predictive performance was achieved in the prostate cohort. We consider the following reasons for this phenomenon: First, this cohort utilized a relatively new hybrid camera system and a high PET target resolution (2.08 × 2.08 × 2.03 mm) and here, reference binary delineations relied on full-mount histopathology slices [30, 49]. However, delineation was still performed on the PET images. This means, that in this cohort, the partial volume effect had the most-significant contribution to the delineation of prostate lesions [50]. Cohorts operating with relatively small lesions are more prone to delineation effects than, for example, binning [51]. This is logical, given, that small lesions are also more prone to the PVE [50, 52] or more sensitive to the absence of point-spread function (PSF) modelling [53]. The PVE was most prominent in our prostate cohort as it had the smallest lesions as well (average lesion volume in prostate: 10.9 cm^3^ vs. 113 cm^3^ in lung and 93 cm^3^ in glioma respectively), where a Ref-F delineation resulted in  +4% cross-validation AUC. This finding was in line with those from Cysouw et al. [53] who investigated the predictive performance of various delineations in [18F]DCFPyL PET-CT prostate patients in combination with analyzing the effect of partial volume correction. The above findings imply that fuzzy radiomics can be ideal to not only handle delineation uncertainties at lesion edges, but to also model partial volume effects directly in the radiomic calculations themselves. Regardless of lesion size, following EARL guidelines and relying on imaging systems operating with FPS modelling has been proven to generally increase radiomic predictive performance in the context of delineation variations [13, 54, 55].

The aggregate performance analysis across the four delineation methods and cohorts within a common lesion volume range revealed that reference fuzzy (Ref-F) delineations in  <35,000 mm^3^ lesions systematically outperformed the reference binary (Ref-B) delineations in all cohorts. While disease-specific imaging characteristics (e.g., infiltrating behavior) may influence these results, it is important to emphasize that all three cohorts were delineated by different clinicians, thus, our findings may also be subjects to interobserver variability bias. This implies that while fuzzy radiomics on its own has added value compared to conventional binary radiomics—especially in small lesions—future studies shall not exclude the analysis of extended fuzzy or binary regions around lesions within their investigations.

While fuzzy radiomics could naturally model a weighted average of multiple clinician-defined delineations, automated approaches have been repeatedly presented as more robust compared to manually-defined delineations that are prone to multi-observer variabilities [13, 14, 43, 56, 57]. In this regard, the study of Hatt et al. [10] investigated a wide-range of automated PET delineation approaches and concluded that while automated approaches have more accurate delineation's compared to simpler manual or semi-automated ones, the potential magnitude of advantage is mainly specific to the given cohort, the scanner and the imaging protocol. Recently, novel deep learning approaches have been reported to provide highly accurate and automated delineation in a wide range of lesion types [13, 58–62]. In the context of automated, especially DL approaches, we wish to emphasize that this study does not promote a particular fuzzy delineation approach, only the concept of incorporating probability weights into standard radiomics calculations. Deep learning is a naturally probabilistic approach; however, its output delineation is routinely post-processed and further dichotomized by a threshold to analyze the lesions by conventional radiomics afterwards [19, 20, 25, 61]. This step introduces an uncertainty into the dichotomized delineation mask [63–65], and overall, results in information loss. Dichotomization does not only influence analyzed lesion boundaries, but may also excludes lesions with relatively lower DL probabilities, that may otherwise be important for predicting the given clinical endpoint. Fuzzy radiomics on the other hand can organically fit the naturally probabilistic output of DL delineation approaches and can minimize the above uncertainties originated by utilizing thresholds.

Further to the above, fuzzy radiomics systematically decreased redundancy across radiomics features in all three involved cohorts by approximately 20%. Due to the naturally high redundancy of various radiomics features [66, 67], they need to undergo redundancy reduction prior to building machine learning models. Redundancy reduction approaches routinely select one from redundant clusters of features having the highest variance [2]. This, however, does not guarantee that the selected feature is the most predictive. Since fuzzy radiomics decreases redundancy, it may support the identification of precise imaging biomarkers in the future by better discriminating features that are otherwise prone to be redundant. Nevertheless, feature redundancy is a phenomenon which is not only affected by inherently similar radiomic calculations, but also by the volume effect [63, 68] which is feature-specific [17, 33]. In this regard, future studies shall investigate how fuzzy radiomics contributes to volume effects, given, that its contribution to decrease feature redundancy is significant.

When looking at the per-delineation feature ranking, a balanced feature rank distribution of high-ranking features was associated to a higher performance which is in line with prior reports [26, 69, 70]. Nevertheless, our aggregated feature ranking analysis suggests that features being high-ranking across multiple delineation types are able to characterize cohort-specific clinical endpoints, regardless of the chosen delineation type. Therefore, we consider such high-ranking features as robust properties of the given cohort to characterize the given clinical endpoint.

According to our findings, we consider that the advantages of fuzzy radiomics are the results of two phenomena: on the one hand, the ability to model imaging system-specific PVE in the radiomic models allows to handle delineation uncertainties, especially in small lesions. On the other hand, the higher number of non-redundant features increases the likelihood of identifying more high-ranking features for building prediction models when relying on fuzzy radiomics.

This study had limitations, namely, that it only utilized single-center cohorts. Nevertheless, the collected cohorts were from different camera systems and relied on various tracers. In addition, this study relied on a high, 100-fold Monte Carlo (MC) cross-validation scheme to estimate the predictive performance of its models built on its delineations and radiomics evaluations in order to minimize the chances of false discoveries. While we employed train-test splits across our MC folds, we relied on mixed ensemble learning to minimize the effects of bias-variance trade-off and we also avoided variations of hyperparameters that could have skewed differences among the four delineation variations.

## Conclusions

Fuzzy radiomics can result in prediction models that outperform conventional binary radiomics-based models, especially in imaging cohorts operating with small lesion sizes. Nevertheless, cohort-specific investigations shall continue to investigate the impact of both fuzzy-vs-binary and lesion-vs-extended lesion volumes in future studies.

## Supplementary Information

Below is the link to the electronic supplementary material.

Supplementary file1 (DOCX 225 KB)

## Data Availability

The datasets generated during and/or analyzed during the current study are available from the corresponding author on reasonable request.
