# Supplementary material for: Machine learning predictive performance evaluation of conventional and fuzzy radiomics in clinical cancer imaging cohorts
Source: Eur J Nucl Med Mol Imaging. 2023 Feb 4;50(6):1607–20. doi: 10.1007/s00259-023-06127-1 (PMC10119059; doi:10.1007/s00259-023-06127-1)
Supplement: Supplementary file 1 — Supplementary file1 (DOCX 225 KB) [file 259_2023_6127_MOESM1_ESM.docx]

**Machine Learning Predictive Performance Evaluation of Conventional and Fuzzy Radiomics in Clinical Cancer Imaging Cohorts**

**SUPPLEMENTAL**

**Patient Cohorts**

In glioma cohort 105 [^11^C]-MET PET images were utilized for this study. The scans were performed between 2000 and 2013. The study was approved by the local institutional review board. Written informed consent was obtained from all patients before the imaging examinations (1,2). Three-years survival as clinical end-point was used to predict in this study. See Supplemental Table 1 for glioma patient characteristics.

**TABLE S1**: Glioma patient characteristics utilized in this study.

| **Patient characteristics** | **Value** | |
| --- | --- | --- |
|  | **n** | **%** |
| **Total patients** | 105 |  |
| **Mean age** ± SD (y) | 47.5 ± 15.36 | |
| **Sex** |  |  |
| Male | 61 | 58.1% |
| Female | 44 | 41.9% |
| **Histologic type** |  |  |
| Diffuse astrocytoma | 36 | 34.3% |
| Oligodendroglioma | 29 | 27.6% |
| Glioblastoma multiforme | 25 | 23.8% |
| Other* | 15 | 14.3% |
| **IDH1 R132H mutation status** |  |  |
| Positive | 49 | 46.7% |
| Negative | 40 | 38.1% |
| Unknown | 16 | 15.2% |

*Other: oligoastrocytoma NOS (WHO 2016), pilocytic astrocytoma and ganglioglioma

In the lung cohort 536 [^18^F]FDG PET/CT images were used from the database of (3). The scans were performed between 2012 and 2019. Ethical approval was obtained and the need for informed consent was waived by the medical ethics committee of the hospital. Two-years survival as clinical end-point was used to predict in this study, where 493 patients had this label available. See Supplemental Table 2 for lung patient characteristics. Tumor stages were defined according to the American Joint Committee on Cancer Staging Manual 7th Edition (4). Histologic growth patterns were based on the 2011 classification of lung adenocarcinoma by the International Association for the Study of Lung Cancer (5). Histologic risk stratification was performed by defining micropapillary (MMP), solid predominant (SPA), and variants of invasive (VIA) lung adenocarcinomas (LUAD) as high-risk; all other growth patterns were defined as low-risk lesions (6–8).

**TABLE S2**: Lung patient characteristics utilized in this study.

| **Patient characteristics** | **Value** | |
| --- | --- | --- |
|  | **n** | **%** |
| **Total patients** | 493 |  |
| **Mean age** ± SD | 60.5 ± 4 |  |
| **Sex** |  |  |
| female | 248 | 50.3% |
| male | 245 | 49.7% |
| **TNM stage** |  |  |
| 0 | 8 | 1.6% |
| 1 | 202 | 41.0% |
| 2 | 18 | 3.7% |
| 3 | 81 | 16.4% |
| 4 | 184 | 37.3% |
| **Growth pattern risk** |  |  |
| low risk | 260 | 52.7% |
| high risk | 76 | 15.4% |
| unknown | 157 | 31.8% |

In prostate cohort 121 lesions from 52 patients having [^68^Ga]Ga-PSMA-11/MRI, collected between 2014 and 2015 were analyzed. The original study was mono-centric pilot study for a prospective randomized trial (clinicaltrials.gov NCT02659527) (9,10). See Supplemental Table 3 for prostate patient characteristics.

**TABLE S3**: Prostate patient and lesion characteristics utilized in this study. IQR – Interquartile range. PSA – Prostate-specific antigen.

| **Patient characteristics (*n* = 52)** | **Value** |
| --- | --- |
| **Age** (years), median (IQR) | 64 (59–70) |
| PSA (ng/ml), median (IQR) | 7.5 (5.0–13.4) |
| **Pathologic T staging, *n* (ratio)** | |
| 2 | 20 (0.38) |
| 2a | 1 (0.02) |
| 2c | 2 (0.04) |
| 3a | 11 (0.21) |
| 3b | 17 (0.33) |
| 4 | 1 (0.02) |
| **Primary Gleason pattern, *n* (ratio)** | |
| 3 | 18 (0.35) |
| 4 | 31 (0.6) |
| 5 | 3 (0.05) |
| **Secondary Gleason pattern, *n* (ratio)** | |
| 3 | 16 (0.31) |
| 4 | 26 (0.5) |
| 5 | 10 (0.19) |
| **Total Gleason Score, *n* (ratio)** | |
| 6 | 3 (0.06) |
| 7 | 14 (0.27) |
| > = 8 | 35 (0.67) |
| **Biochemical recurrence (BCR), *n* (ratio)** | |
| Yes | 9 (0.17) |
| No | 27 (0.52) |
| NA | 16 (0.31) |
| **Follow-up (months), median (IQR)** | **41 (32–49)** |
| **Characteristics of the 121 delineated lesions in the 52 patients** | |
| **Lesion characteristics (*n* = 121)** | **Value** |
| **Delineated lesions, *n* (ratio)** | |
| Benign prostatic hyperplasia | 20 (0.17) |
| Low grade PIN | 16 (0.13) |
| High grade PIN | 5 (0.04) |
| Prostatitis | 2 (0.02) |
| Gleason 3 | 17 (0.14) |
| Gleason 4 | 50 (0.41) |
| Gleason 5 | 11 (0.09) |
| **Lesion high-low risk pattern, *n* (ratio)** | |
| High risk pattern | 61 (0.504) |
| Low risk pattern | 60 (0.496) |

See details of the imaging protocols as well as the IBSI radiomics feature extraction parameters in the IBSI-conform Supplemental Table 4.

**IBSI Radiomics**

**TABLE S4**: IBSI reporting structure of the study. The information presented herein is based on the Imaging Biomarker Standardization Initiative (IBSI) guidelines (11).

| **Glioma Patient group** | |
| --- | --- |
| Volume of Interest | [^11^C]-MET-positive lesions in glioma |
| Patient Preparation | Patients were required to fast for at least 5 h before injection of 770 ± 106 MBq ^11^C-MET based on body weight. |
| Radiotracer | [^11^C]-MET [^11^C]-Methionine |
| Protocol | A dedicated glioma PET scan was performed over one PET bed position with patients in prone position. |
| Scanner type | Advance PET system (GE Healthcare) |
| 18C-MET | 770 ± 106 MBq injected  10 min emission time  5 min transmission  20 min uptake time  128 × 128 axial matrix size  3.6 × 3.6 × 5 mm voxel size  5 mm physical resolution (10cm) – as of hard cover book "Technical Publication Direction 2103139-100 Revision 4, Advance PET imaging System Description And Specification (Service Documentation)" |

| **Lung Patient group** | | |
| --- | --- | --- |
| Volume of Interest | [¹⁸F]FDG-positive thoracic lung adenocarcinoma lesions in PET/CT | |
| Patient Preparation | Fasting 6h prior tracer injection  Blood glucose measurement (required < 200mg/dl) | |
| Radiotracer | [¹⁸F]FDG (>90% radiochemical purity)  5.55 MBq/kg (0,15 mCi/kg) [¹⁸F]FDG injected intravenously  60 minutes uptake time resting recumbent in a calm environment |  |
| Protocol | Local protocol of 3. Hospital of Peking Medical University – Department of Nuclear Medicine | |
| Scanner type | Siemens Biograph TruePoint PET/CT | |
| [¹⁸F]FDG-PET | Static  5-7 bed positions  2-2,5 minutes per bed position  2.98 mm voxel size  Matrix size: 168x168  Slice thickness: 3 mm  Image slice spacing 0 mm  Reconstruction: OSEM (21 subsets, 3 iterations)  Scatter and attenuation correction: CT based 4.7 mm physical resolution (10cm) – as of <https://meditegic.com/wp-content/uploads/pdfs/Siemens_Biograph.pdf> | |

| CT | Deep inspiratory CT with breath holding  Tube voltage: 120 kVp  Tube current: 100 mAs  0.98 mm voxel size  Matrix size: 512x512  Slice thickness: 3 mm |
| --- | --- |

| **Prostate Patient Group** | |
| --- | --- |
| Volume of Interest | [^68^Ga]Ga-PSMA-11 PET/MRI-positive lesions in prostate |
| Patient Preparation | As of clinicaltrials.gov NCT02659527 |
| Radiotracer | [^68^Ga]Ga-PSMA-11 |
| Protocol | As of clinicaltrials.gov NCT02659527 |
| Scanner type | Siemens Biograph 3T PET/MRI |
| [^68^Ga]Ga-PSMA-11 | Static (the last 10 min of dynamic scan)  45 min after [^18^F]FMC (dual-tracer acquisition)  2.08 x 2.08 x 2.03mm voxel size  4.6 mm physical resolution (10cm) as of <https://www.siemens-healthineers.com/at/magnetic-resonance-imaging/mr-pet-scanner/biograph-mmr> |
| T2w | Static  TR - 750, TE - 109 and FA-100  0.9 x 0.9 x 0.9 mm voxel size |
| ADC | Static  TR - 5300 and TE – 92  1.2 x 1.2 x 3.6 mm voxel size |
| TR – time of relaxation, TE – time of echo, FA – flip angle, ADC – Apparent Diffusion Coefficient | |

| **Settings of all Cohorts** | |
| --- | --- |
| **Image Co-registration** | |
| Software | Hermes Hybrid 3D ver 4.0.0 |
| Co-registration step | Automated as of DICOM coordinate parameters |
| **Data conversion** | |
| Step 1 (all images) | Initial voxel values determined by transforming the DICOM raw voxel values with the DICOM tags Rescale Scope (0028\|1053) and Rescale Intercept (0028\|1052). |
| Step 2 (PET) | Initial voxel values transformed to tumor-to-background ratio (TBR) by dividing all voxel values with the mean of the reference region drawn as a 4 × 4 × 4 cuboid VOI in a non-affected background region in each patient. Background regions were cohort-specific: contralateral (Glioma), mediastinum (Lung), gluteus maximus (Prostate) |
| **Segmentation** | |
| Software | Hermes Hybrid 3D ver 4.0.0 |
| VOI definition | Standard semi-automated iso-count 3D VOI tools |
| Number of experts | 1 + 1 (1 nuclear medicine expert participated in independent delineations, followed by 1 senior nuclear medicine specialist cross-validation and if necessary, modification of first-round results) for glioma and lung, 4 + 1 for prostate (4 nuclear medicine expert participated in independent delineations, followed by 1 senior nuclear medicine specialist cross-validation and if necessary, modification of first-round results) |
| Reference image | PET |
| **Image / VOI interpolation** | |
| Method | Kriging interpolation in 3D, including nearest neighbors in distance of voxel size main diagonal (12). |
| Grid | Align by center |
| Extrapolation beyond original image | Neighbor distance search calculated as original voxel size main diagonal + epsilon. Missing value: image minimum |
| Voxel dimensions | 2.0 mm uniform voxel sizes as of (13) |
| Partially masked voxels (VOI) | Taken if more than half of original voxel area included |
| **Discretization** | |
| Method | Fixed bin width, variable number of bins |
| Bin width | 0.05 (PET) |
| **Image biomarker computation / Parameters** | |
| **Intensity-based statistical features** (22 per image): Mean intensity, Intensity variance, Intensity skewness, (Excess) intensity kurtosis, Median intensity, Minimum intensity, 10th intensity percentile, 90th intensity percentile, Maximum intensity, Intensity interquartile range, Intensity range, Intensity-based mean absolute deviation, Intensity-based robust mean absolute deviation, Intensity-based median absolute deviation, Intensity-based coefficient of variation, Intensity-based quartile coefficient of dispersion, Intensity-based energy, Root mean square intensity, Local intensity peak, Global intensity peak , Intensity sum  **GLCM features** (24 per image): Joint maximum, Joint average, Joint variance, Joint entropy, Difference variance, Difference entropy, Sum average, Sum variance, Sum entropy, Angular second moment, Contrast, Dissimilarity, Inverse difference, Normalised inverse difference, Inverse difference moment, Normalised inverse difference moment, Inverse variance, Correlation, Autocorrelation, Cluster shade, Cluster prominence, Information correlation 1, Information correlation 2, Difference average  **GLSZM features** (16 per image): Small zone emphasis, Large zone emphasis, Low grey level zone emphasis, High grey level zone emphasis, Small zone low grey level emphasis, Small zone high grey level emphasis, Large zone low grey level emphasis, Grey level non-uniformity, Normalised grey level non-uniformity, Zone size non-uniformity, Normalised zone size non-uniformity, Zone percentage, Grey level variance, Zone size variance, Zone size entropy, Large zone high grey level emphasis  **Intensity histogram features** (19 per image): Mean discretised intensity, Discretised intensity variance, Discretised intensity skewness, (Excess) discretised intensity kurtosis, Median discretised intensity, Minimum discretised intensity, Maximum discretised intensity, Intensity histogram mode, Intensity histogram mean absolute deviation, Intensity histogram robust mean absolute deviation, Intensity histogram median absolute deviation, Intensity histogram coefficient of variation, Intensity histogram quartile coefficient of dispersion, Discretised intensity entropy, Discretised intensity uniformity, Maximum histogram gradient, Maximum histogram gradient intensity, Minimum histogram gradient, Minimum histogram gradient intensity  **Intensity-volume histogram features** (6 per image): Volume at intensity fraction 10, Volume at intensity fraction 90, Intensity at volume fraction 10, Intensity at volume fraction 90, Volume fraction difference between intensity fractions, Intensity fraction difference between volume fractions  **Morphological features** (12 per image): Volume (mesh), Surface area (mesh), Surface to volume ratio, Volume (voxel counting), Compactness 1, Compactness 2, Spherical disproportion, Sphericity, Asphericity, Centre of mass shift, Volume density (axis-aligned bounding box), Area density (axis-aligned bounding box)  **NGTDM features** (5 per image): Coarseness, Contrast, Busyness, Complexity, Strength  **GLRLM features** (16 per image): Short run low grey level emphasis, Short run high grey level emphasis, Long run low grey level emphasis, Long run high grey level emphasis, Grey level variance, Run length variance, Run entropy, Short runs emphasis, Long runs emphasis, Low grey level run emphasis, High grey level run emphasis, Grey level non-uniformity, Normalised grey level non-uniformity, Run length non-uniformity, Normalised run length non-uniformity, Run percentage  **NGLDM features** (17 per image): Low dependence low grey level emphasis, Low dependence high grey level emphasis, High dependence low grey level emphasis, High dependence high grey level emphasis, Dependence count percentage, Grey level variance, Dependence count variance, Dependence count entropy, Dependence count energy, Low dependence emphasis, High dependence emphasis, Low grey level count emphasis, High grey level count emphasis, Grey level non-uniformity, Normalised grey level non-uniformity, Dependence count non-uniformity, Normalised dependence count non-uniformity  **GLDZM features** (16 per image): Small distance emphasis, Large distance emphasis, Low grey level zone emphasis, High grey level zone emphasis, Small distance low grey level emphasis, Small distance high grey level, emphasis, Large distance low grey level emphasis, Large distance high grey level emphasis, Grey level non-uniformity, Normalised grey level non-uniformity, Grey level variance, Zone distance non-uniformity, Normalised zone distance non-uniformity, Zone distance variance, Zone distance entropy, Zone percentage | |
| Software | MUW radiomics engine (13). Software availability upon request from the corresponding author. |
| Distance weighting | No |
| CM symmetry | Symmetric |
| CM / ZM distance | Chebyshev distance 1 |
| CM / ZM aggregation | 3D, full-merging |
| Exclusion criteria | VOIs with less than 64 voxels were excluded from the analysis |

**Fuzzy vs. Classic Radiomics**

Fuzzy Radiomics has been proposed and demonstrated on physical PET phantoms by Papp et al (14). The concept of fuzzy radiomics only differs from classic radiomics in the way it handles delineation masks that are inputs of radiomic analyses next to the given image to analyze. In general, classic radiomic calculations require an input image and a respective binary mask as input. The binary mask determines which voxels from the given image are subjects of the analysis.

Fuzzy radiomics generalizes the above approach by allowing not only 1.0 and 0.0 values in the given mask, but also any value in-between (see Figure S1).

Once any intermediate data containers are filled in, radiomic feature extraction is taken place by executing a set of equations for the given data container as defined in the IBSI guideline (11). According to the above, fuzzy and classic radiomics both operate with identical IBSI equations. Hence, classic radiomics is a special variant of fuzzy radiomics. Specifically, in case a fuzzy radiomic engine gets a probability mask as input having only 0.0 and 1.0 values, the given analysis outcome will be identical to classic IBSI-conform radiomics.

Note that fuzzy radiomics does not promote any particular delineation method which provides a fuzzy probability mask for its calculations, it merely assumes that it gets such as probability mask as input.

**
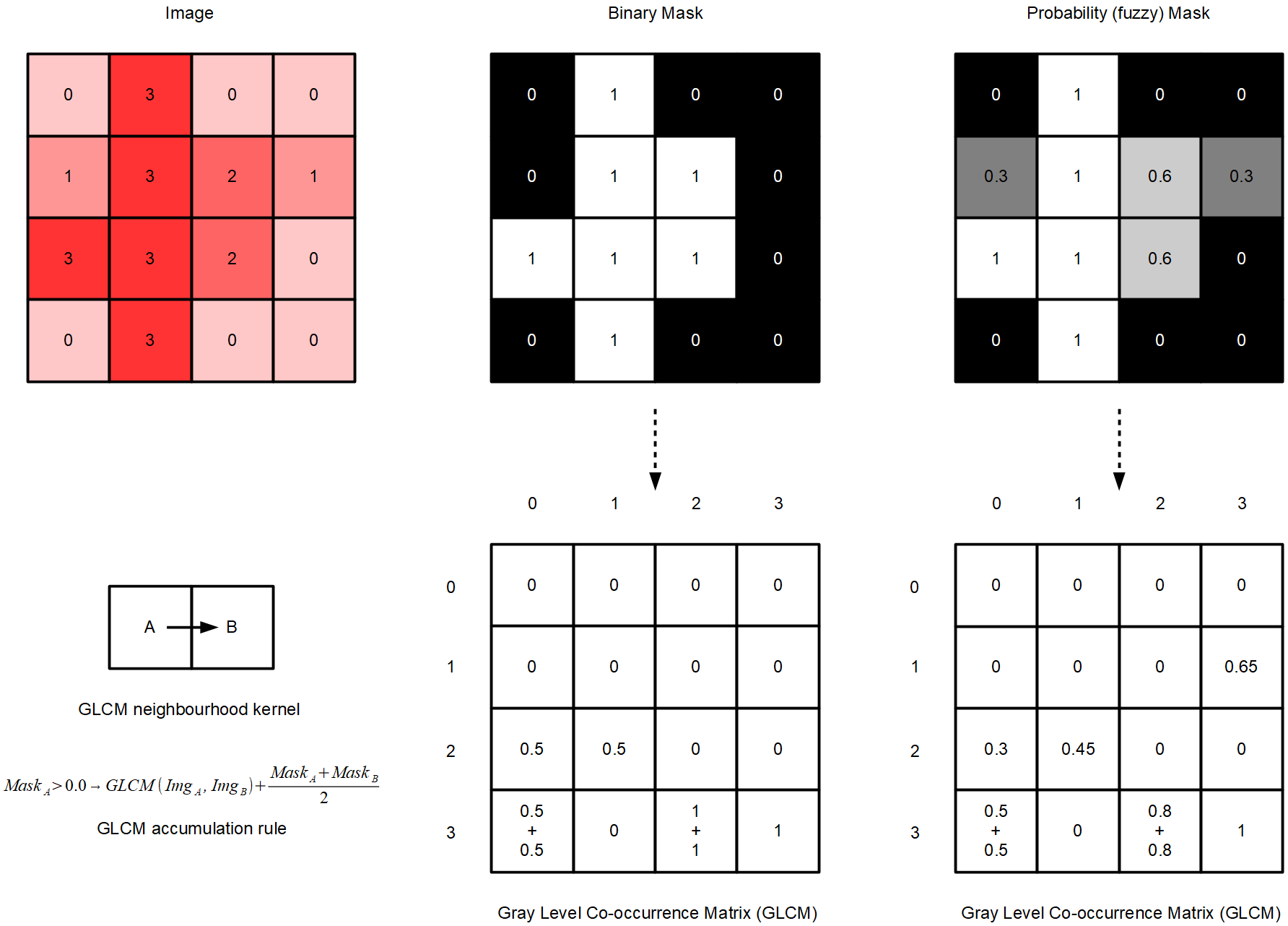
**

**Figure S1:** Demonstration of differences between classic and fuzzy radiomics. Given a 2D image (Img), it has values between 0-3. The respective binary mask contains 0 or 1 values, while the probability (fuzzy) mask contains values in-between 0.0 – 1.0. In this example, the gray-level co-occurrence matrix (GLCM) neighborhood kernel only considers left-to-right neighbors. The GLCM accumulation rule is defined as the average value of the given mask of left-to-right neighbors in the given image, but the GLCM is only updated if voxel value A has a >0.0 mask value. Note the differences in the binary and fuzzy mask-generated GLCM entries, originated from the differences between binary and fuzzy masks.

**Machine learning predictive models**

Four random forest algorithms and one genetic algorithm (GA) (1,9) variants (5 total) were employed in an ensemble learning scheme. The final model decision was obtained by averaging across the five predictive models (see TABLE S5).

**TABLE S5**: Random Forest (RF) algorithms settings of the 4 RF models and the genetic algorithm (GA) settings of the 1 GA model employed in the ensemble learning scheme (1). KDE – Kernel Density Estimation (15).

| **Parameter** | **RF-1** | **RF-2** | **RF-3** | **RF-4** | **Parameter** | **GA-1** |
| --- | --- | --- | --- | --- | --- | --- |
| Number of trees | 100 | 400 | 300 | 300 | Tolerance | 0.00025 |
| Quality metric | gain | | | | Maximum iteration count | 10 |
| Max depth | 5 | 10 | 10 | 15 |  |  |
| Min samples at leaf | 5 | 5 | 5 | 3 | Population count per iteration | 20 |
| Feature selection | Random | | | |  |  |
| KDE attributes per split | 10 | 15 | 20 | 25 | Maximum mutation rate | 0.5 |
| Random features | 10 | 6 | 4 | 4 |  |  |
| Number selected trees | 10 | | | | Minimum mutation rate | 0.1 |
| Bagging method | equalized | | | |  |  |
| Bag fraction | 1.0 | | | |  |  |

**Feature Ranking**

The following figures (Figure S2 – 13) demonstrate the feature ranking and selection occurrences across each cohort's 100-fold Monte Carlo cross-validation folds with the four delineation approaches as provided by the Genetic Algorithm (GA), which determined feature weights during its training processes. Ranks are normalized to the sum of 1.0.

**Glioma patient group**

**
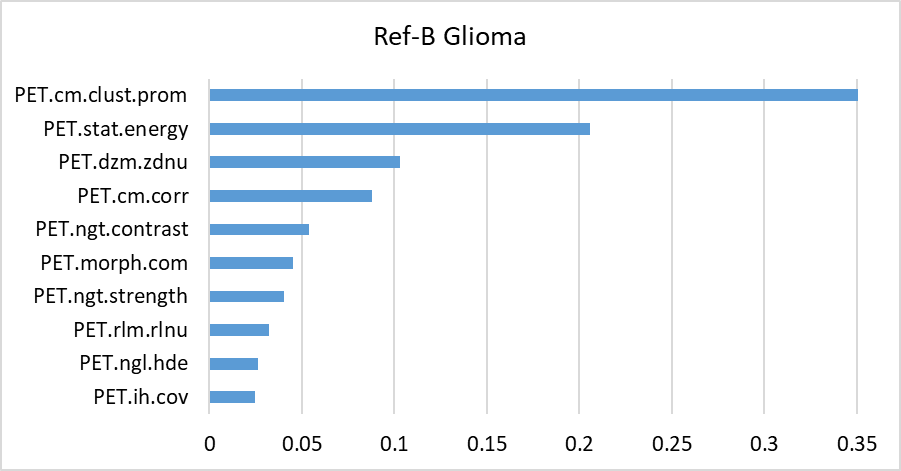
**

**Figure S2**: Occurrences of the most prominent 10 features across 100 Monte Carlo folds in the Glioma cohort relying on the Reference Binary (Ref-B) delineation approach.


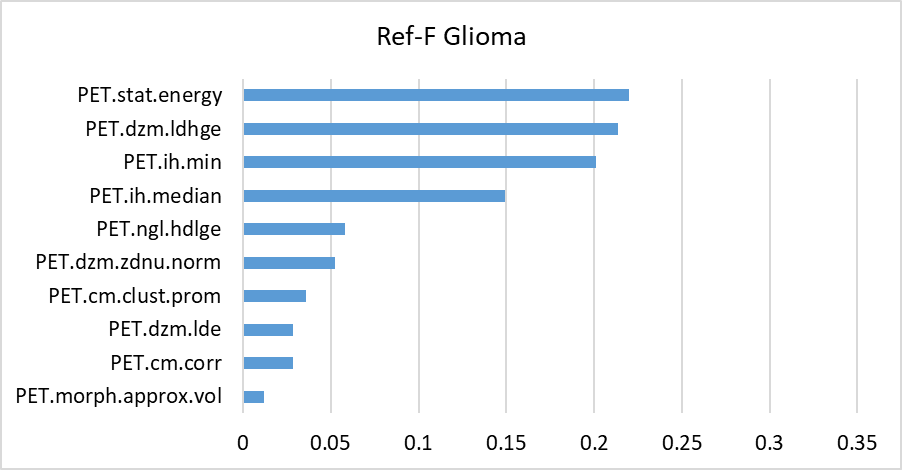


**Figure S3**: Occurrences of the most prominent 10 features across 100 Monte Carlo folds in the Glioma cohort relying on the Reference Fuzzy (Ref-F) delineation approach.


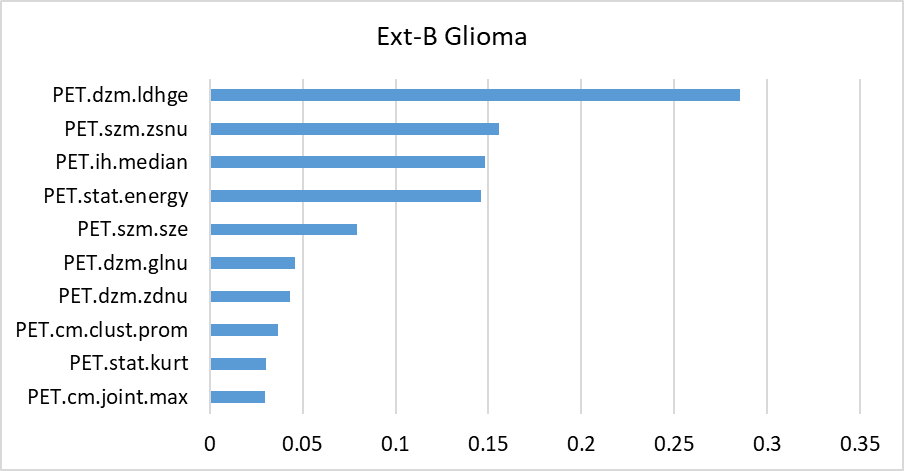


**Figure S4**: Occurrences of the most prominent 10 features across 100 Monte Carlo folds in the Glioma cohort relying on the Extended Binary (Ext-B) delineation approach.


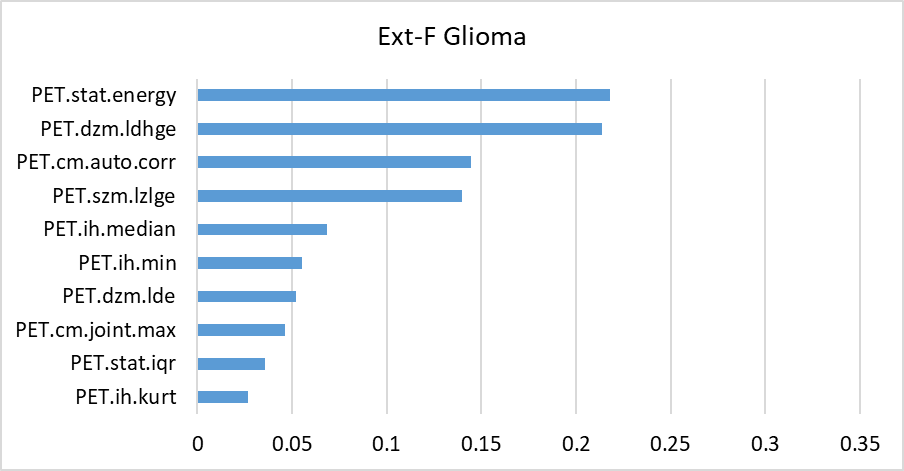


**Figure S5**: Occurrences of the most prominent 10 features across 100 Monte Carlo folds in the Glioma cohort relying on the Extended Fuzzy (Ext-F) delineation approach.

**Lung patient group**


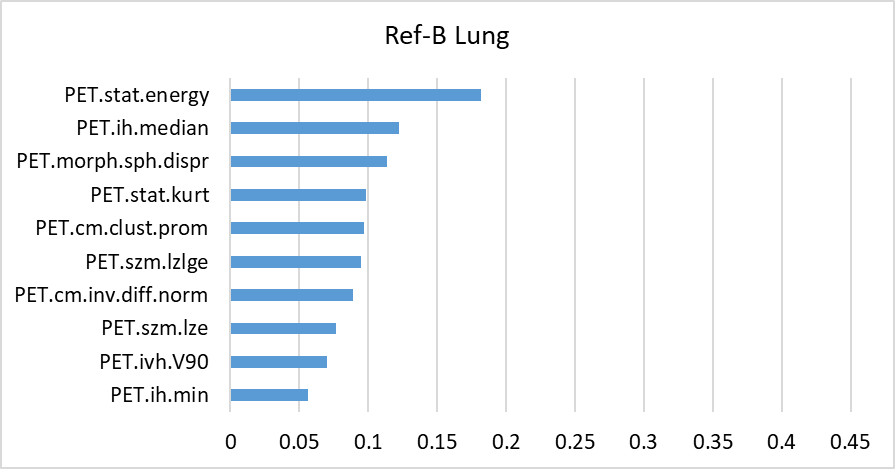


**Figure S6**: Occurrences of the most prominent 10 features across 100 Monte Carlo folds in the Lung cohort relying on the Reference Binary (Ref-B) delineation approach.


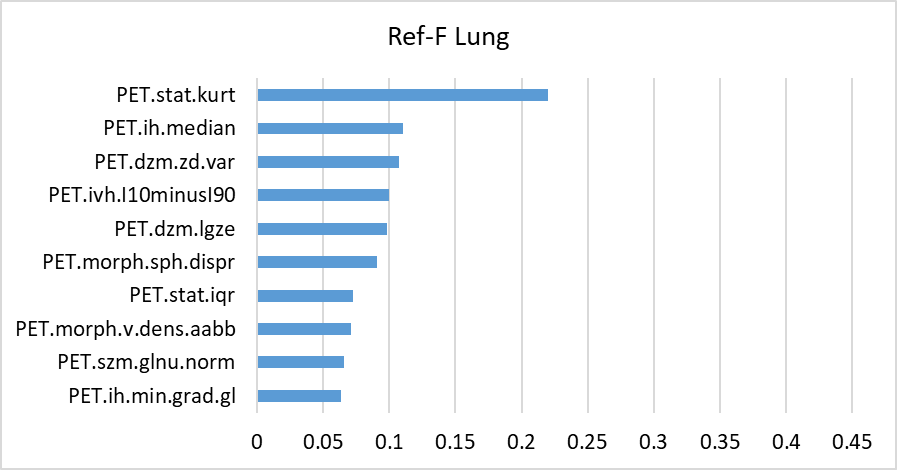


**Figure S7**: Occurrences of the most prominent 10 features across 100 Monte Carlo folds in the Lung cohort relying on the Reference Fuzzy (Ref-F) delineation approach.


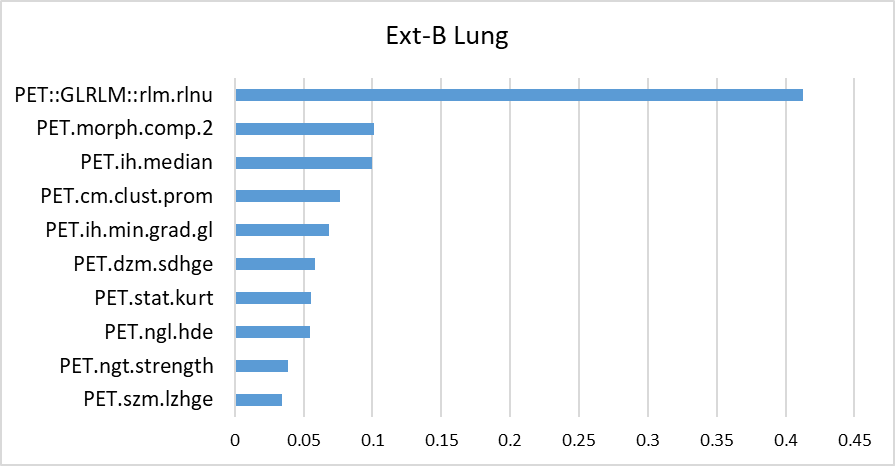


**Figure S8**: Occurrences of the most prominent 10 features across 100 Monte Carlo folds in the Lung cohort relying on the Extended Binary (Ext-B) delineation approach.


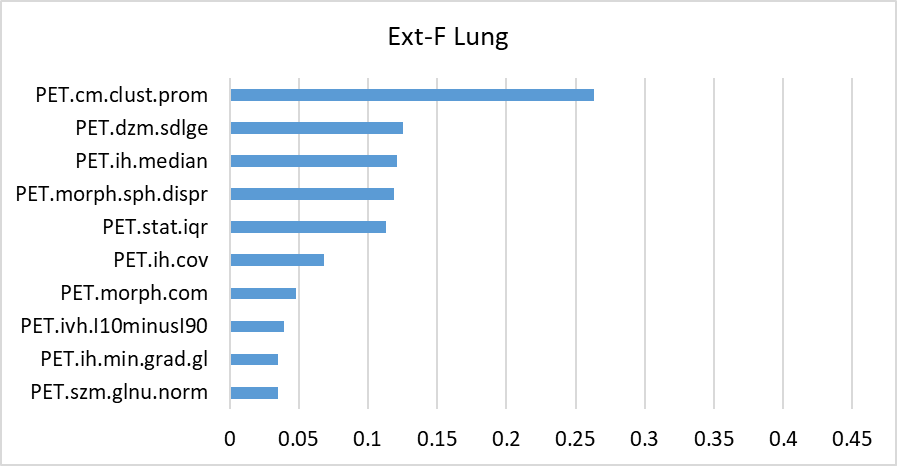


**Figure S9**: Occurrences of the most prominent 10 features across 100 Monte Carlo folds in the Lung cohort relying on the Extended Fuzzy (Ext-F) delineation approach.

**Prostate patient group**


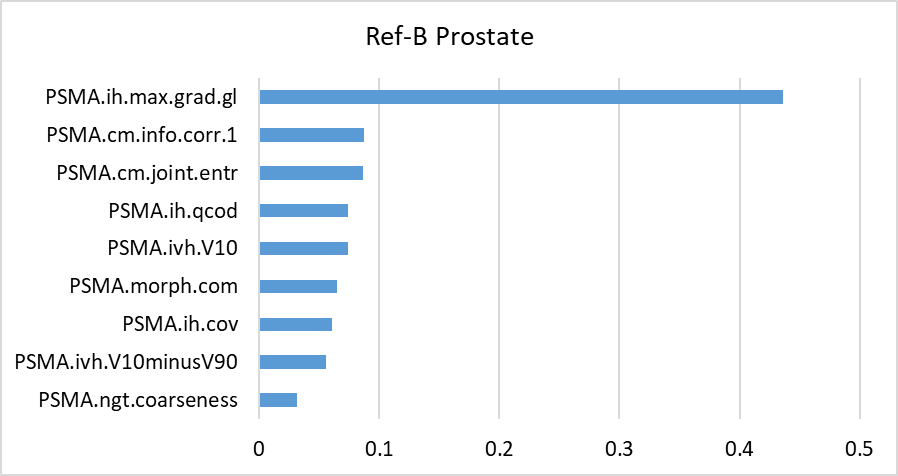


**Figure S10**: Occurrences of the most prominent 10 features across 100 Monte Carlo folds in the Prostate cohort relying on the Reference Binary (Ref-B) delineation approach.


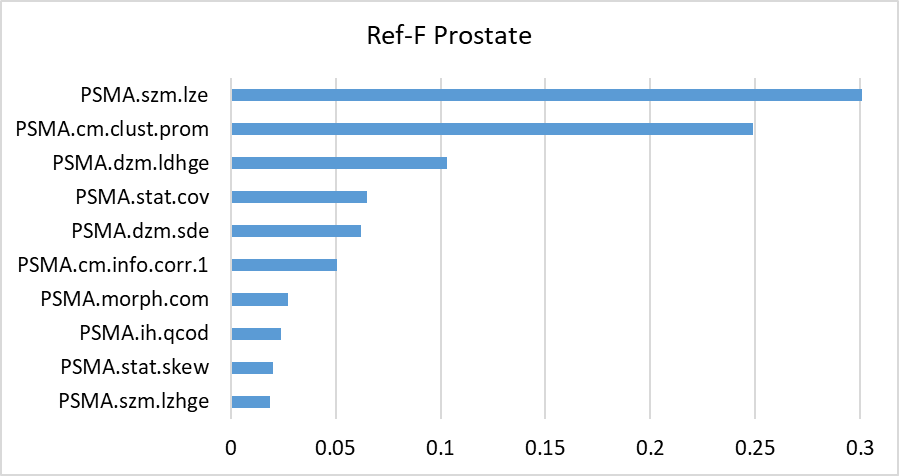


**Figure S11**: Occurrences of the most prominent 10 features across 100 Monte Carlo folds in the Prostate cohort relying on the Reference Fuzzy (Ref-F) delineation approach.


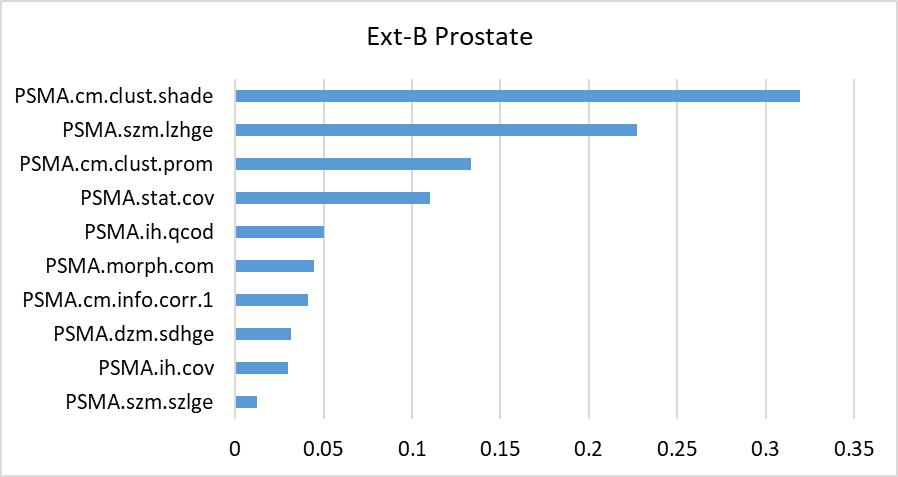


**Figure S12**: Occurrences of the most prominent 10 features across 100 Monte Carlo folds in the Prostate cohort relying on the Extended Binary (Ext-B) delineation approach.


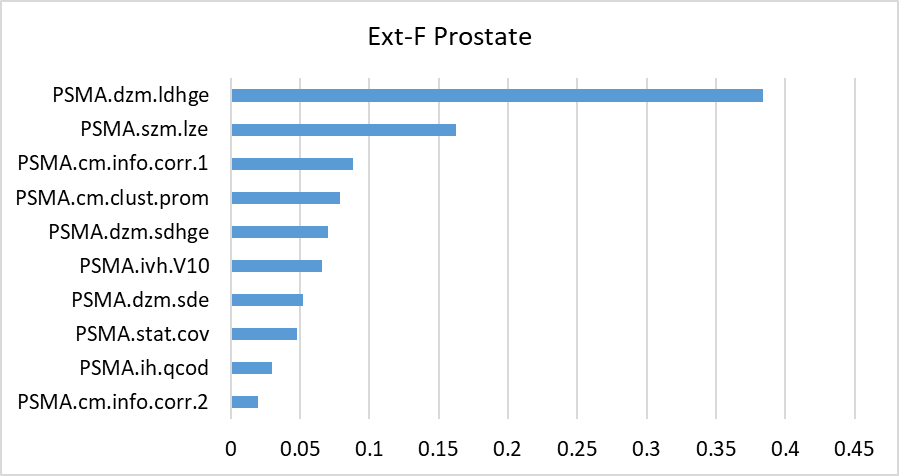


**Figure S13**: Occurrences of the most prominent 10 features across 100 Monte Carlo folds in the Prostate cohort relying on the Extended Fuzzy (Ext-F) delineation approach.

**References**

1. Papp L, Poetsch N, Grahovac M, Schmidbauer V, Woehrer A, Preusser M, et al. Glioma survival prediction with the combined analysis of in vivo 11C-MET-PET, ex vivo and patient features by supervised machine learning. J Nucl Med [Internet]. 2017;59(6):jnumed.117.202267. Available from: http://jnm.snmjournals.org/lookup/doi/10.2967/jnumed.117.202267

2. Poetsch N, Woehrer A, Gesperger J, Furtner J, Haug AR, Wilhelm D, et al. Visual and semiquantitative 11C-methionine PET: an independent prognostic factor for survival of newly diagnosed and treatment-naïve gliomas. Neuro Oncol [Internet]. 2018 Feb 19;20(3):411–9. Available from: https://academic.oup.com/neuro-oncology/article/20/3/411/4110230

3. Zhao M, Kluge K, Papp L, Grahovac M, Yang S, Jiang C, et al. Multi-lesion radiomics of PET/CT for non-invasive survival stratification and histologic tumor risk profiling in patients with lung adenocarcinoma. Eur Radiol [Internet]. 2022 Jul 28;32(10):7056–67. Available from: https://link.springer.com/10.1007/s00330-022-08999-7

4. Edge SB, Compton CC. The American Joint Committee on Cancer: the 7th Edition of the AJCC Cancer Staging Manual and the Future of TNM. Ann Surg Oncol [Internet]. 2010 Jun 24;17(6):1471–4. Available from: http://link.springer.com/10.1245/s10434-010-0985-4

5. Travis WD, Brambilla E, Noguchi M, Nicholson AG, Geisinger KR, Yatabe Y, et al. International Association for the Study of Lung Cancer/American Thoracic Society/European Respiratory Society International Multidisciplinary Classification of Lung Adenocarcinoma. J Thorac Oncol [Internet]. 2011 Feb;6(2):244–85. Available from: https://linkinghub.elsevier.com/retrieve/pii/S1556086415319304

6. Yoshizawa A, Motoi N, Riely GJ, Sima CS, Gerald WL, Kris MG, et al. Impact of proposed IASLC/ATS/ERS classification of lung adenocarcinoma: prognostic subgroups and implications for further revision of staging based on analysis of 514 stage I cases. Mod Pathol [Internet]. 2011 May;24(5):653–64. Available from: https://linkinghub.elsevier.com/retrieve/pii/S089339522202871X

7. Strand T-E, Rostad H, Strøm EH, Hasleton P. The percentage of lepidic growth is an independent prognostic factor in invasive adenocarcinoma of the lung. Diagn Pathol [Internet]. 2015 Dec 9;10(1):94. Available from: https://diagnosticpathology.biomedcentral.com/articles/10.1186/s13000-015-0335-8

8. Li H, Cao W. Pulmonary enteric adenocarcinoma: a literature review. J Thorac Dis [Internet]. 2020 Jun;12(6):3217–26. Available from: http://jtd.amegroups.com/article/view/40341/html

9. Papp L, Spielvogel CP, Grubmüller B, Grahovac M, Krajnc D, Ecsedi B, et al. Supervised machine learning enables non-invasive lesion characterization in primary prostate cancer with [68Ga]Ga-PSMA-11 PET/MRI. Eur J Nucl Med Mol Imaging [Internet]. 2020 Dec 19; Available from: http://link.springer.com/10.1007/s00259-020-05140-y

10. Hartenbach M, Hartenbach S, Bechtloff W, Danz B, Kraft K, Klemenz B, et al. Combined PET/MRI improves diagnostic accuracy in patients with prostate cancer: A prospective diagnostic trial. Clin Cancer Res [Internet]. 2014 Jun 15;20(12):3244–53. Available from: http://clincancerres.aacrjournals.org/cgi/doi/10.1158/1078-0432.CCR-13-2653

11. Zwanenburg A, Leger S, Vallières M, Löck S, Initiative for the IBS. Image biomarker standardisation initiative. arXiv [Internet]. 2016;(November). Available from: http://arxiv.org/abs/1612.07003

12. Stytz MR, Parrott RW. Using kriging for 3d medical imaging. Comput Med Imaging Graph. 1993;17(6):421–42.

13. Papp L, Rausch I, Grahovac M, Hacker M, Beyer T. Optimized Feature Extraction for Radiomics Analysis of 18 F-FDG PET Imaging. J Nucl Med [Internet]. 2019 Jun;60(6):864–72. Available from: http://jnm.snmjournals.org/lookup/doi/10.2967/jnumed.118.217612

14. Papp L, Rausch I, Hacker M, Beyer T. Fuzzy Radiomics: A novel approach to minimize the effects of target delineation on radiomic models. In 2019. Available from: http://www.thieme-connect.de/DOI/DOI?10.1055/s-0039-1683478

15. Geng X, Hu G. Unsupervised feature selection by kernel density estimation in wavelet-based spike sorting. Biomed Signal Process Control [Internet]. 2012 Mar 1 [cited 2017 Oct 17];7(2):112–7. Available from: http://www.sciencedirect.com/science/article/pii/S1746809411000279
